# Extracellular Vesicles in Head and Neck Cancer: A Potential New Trend in Diagnosis, Prognosis, and Treatment

**DOI:** 10.3390/ijms21218260

**Published:** 2020-11-04

**Authors:** Xinyu Qu, Jing-Woei Li, Jason Chan, Katie Meehan

**Affiliations:** 1 Department of Otorhinolaryngology, Head and Neck Surgery, Faculty of Medicine, The Chinese University of Hong Kong, Hong Kong, China; xyqu@surgery.cuhk.edu.hk (X.Q.); jasonchan@ent.cuhk.edu.hk (J.C.); 2 Department of Ear, Nose and Throat, Queen Elizabeth Hospital, Hong Kong, China; marcoli@link.cuhk.edu.hk; 3 Department of Surgery, Queen Elizabeth Hospital, Hong Kong, China

**Keywords:** extracellular vesicles, head and neck cancer, diagnosis, therapy

## Abstract

Head and neck cancer (HNC) is a fatal and debilitating disease that is characterized by steady, poor survival rates despite advances in treatment. There is an urgent and unmet need to improve our understanding of what drives this insidious cancer and causes poor outcomes. Extracellular vesicles (EVs) are small vesicles that originate from tumor cells, immune cells, and other cell types and are secreted into plasma, saliva, and other bio-fluids. EVs represent dynamic, real-time changes of cells and offer an exciting opportunity to improve our understanding of HNC biology that may translate to improved clinical practice. Considering the amplified interest in EVs, we have sought to provide a contemporary review of the most recent and salient literature that is shaping the field. Herein, we discuss the functionality of EVs in HNCs and their clinical potential with regards to biomarker and therapeutic capabilities.

## 1. Introduction

**Figure 1:**
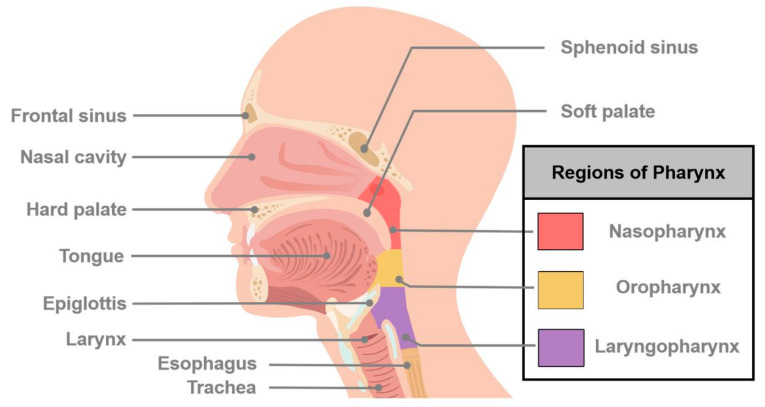
Schematic diagram of major sites of cancers of head and neck. Head and neck cancer (HNC) is a general term that describes many different types of cancers and involves multiple organs and tissues within the head and neck region. HNCs generally occur in the oral cavity, oropharynx, hypopharynx, nasopharynx, larynx, and nasal cavity.

Cancers of the head and neck (HNCs) are predominantly squamous cell carcinoma (SCC) and arise in a variety of anatomical sites including the oral cavity, nasopharynx, oropharynx, hypopharynx, larynx, and others (Figure 1) [1]. They are the sixth most common malignancy worldwide with over 800,000 new cases and 400,000 deaths annually and account for over 3.6% (65,360 cases) of new cases of all cancers and over 2.4% (14,500 deaths) of cancer deaths in the United States alone [2,3]. Important global risk factors and oncogenic drivers include long-term tobacco use, high alcohol consumption, and viral infection (namely the Human Papilloma Virus or Epstein-Barr Virus) [4,5]. Despite some advances in therapeutic approaches over the past few decades, most HNCs are treated similarly with a combination of surgery, radio- and/or chemotherapy. Unfortunately, there has been incremental progress in prolonging survival even with the advent of targeted and immunotherapies.

Most HNCs originate deep within the nasal cavity, oral cavity, pharynx, or larynx and are not easily palpated, inspected, or realized by patients. Consequently, approximately 50% of cases are diagnosed with advanced disease and succumb to their illness within 5 years. The need for a reliable non-invasive biomarker is highlighted by studies that have shown an improvement of 5-year survival rates to 80% when HNC is detected at an early stage [6]. Currently, tumor tissue and serial needle biopsies are widely performed for the diagnosis and prognostication of HNCs but have important limitations. Beyond their invasive nature, discrete tissue biopsies cannot show the complete picture of a tumor due to inter- and intra-tumoral heterogeneity and the dynamic tumor microenvironment. As an alternative, liquid biopsies are being explored as a real-time way to measure disease. While studies have established the clinical significance of cell-free DNA for minimally invasive diagnosis of HNCs (in particular nasopharyngeal cancer), the translational potential of EVs is yet to be realized. This review has explored the current knowledge of EVs in HNC biology and precision medicine.

## 2. Extracellular Vesicles

**Figure 2:**
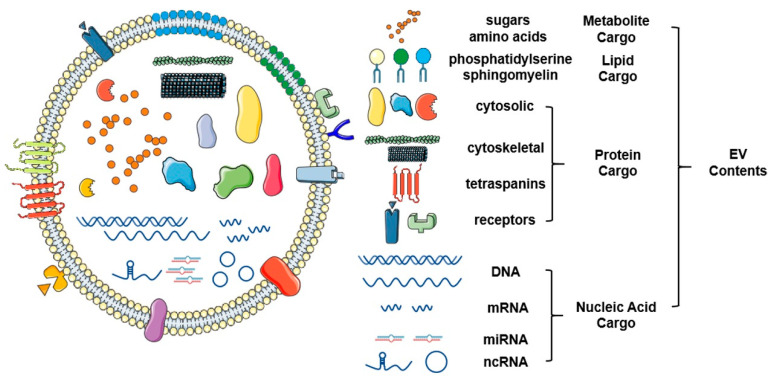
Schematic representation of the structure and contents of extracellular vesicles. Extracellular vesicles are a class of small vesicles encapsulated with a bilayer of lipid that surrounds a variety of functional molecular contents, such as proteins, nucleic acids, and lipids. As novel omics technologies continue to develop, a large scale of screening and identification of extracellular vesicles (EV) contents will continue to mature. This figure was created with Smart Servier (https://smart.servier.com) and BioRender.com.

**Figure 3:**
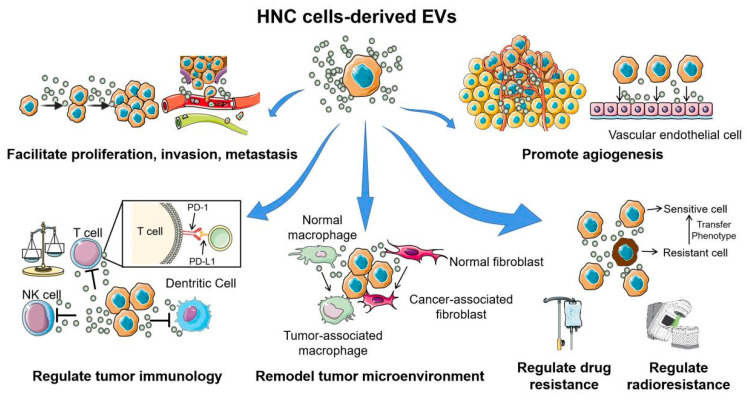
The multiple functions of extracellular vesicles in head and neck cancer carcinogenesis and development. Head and neck cancer cell-derived EVs are involved in almost all hallmarks of cancer development and progression. Through the horizontal transfer of molecular contents to the surrounding tumor cells, immune cells, and stromal cells, EVs play important roles in facilitating proliferation, invasion, metastasis, angiogenesis, conferring chemo- or radio-resistant phenotypes, and help form a “tumor-friendly” environment. This figure was created with Smart Servier (https://smart.servier.com) and BioRender.com.

EVs are functional nanovesicles that are deliberately packaged and secreted by physiologically normal and pathological cells. They are enclosed by natural lipid bilayers and segments of cell membranes that are clipped off during EV formation [7]. EVs harbor molecular cargo that directly reflects their cell of origin and are increasingly recognized as major drivers of cell-cell communication (Figure 2) [8]. These small packages specifically target recipient cells and relay signals via cell surface receptors and/or transfer of their contents directly and deliberately. Their abilities to protect their molecular cargo and provide a safe mode of transport to distant sites throughout the body underscore their importance in carcinogenesis and tumor development (Figure 3).

EV research is no longer in its infancy and the complexity of EV heterogeneity is important. We have used the most recent guidelines outlined in the minimal information for studies of extracellular vesicles position statement [9] to assign the descriptor “EVs” where appropriate. Further, we have included operational terms for EV subtypes (e.g., small EVs, medium/large EVs, CD63^+^/CD81^+^ EVs, apoptotic bodies) where possible. We have used the term extracellular particles (EPs) for data presented by studies that have not adequately addressed the minimal MISEV2018. Specific details regarding these criteria for each study are included in the Supplementary Table S1.

### 2.1. EVs Facilitate Proliferation, Invasion, and Metastasis

Lymph node involvement and distant metastasis are common among HNC patients and are the leading causes of treatment failure and death. Epithelial to mesenchymal transition (EMT) is considered an important feature of tumor progression that facilitates tumor cell motility and invasion into the stroma. Matrix metalloproteinases (MMP) are important enzymes that remodel the extracellular matrix and can lead to decreased cell adhesion and enhanced invasion and metastasis. Considering this, it is not surprising that MMP13 is overexpressed in nasopharyngeal cancer (NPC) cells, EPs from NPC cells, and in plasma of patients with NPC [10]. Functional studies using crudely isolated and minimally characterized EPs hinted that MMP13^+^ EPs from the plasma of patients with NPC may mediate the tumor microenvironment by facilitating tumor cell migration and invasion via promoting interaction with stromal cells. However, soluble factors within plasma may also mediate these activities and additional studies are needed that include appropriate controls before these functions can be accurately attributed to EPs. Additionally, EPs from NPC cells cultured in hypoxic conditions were significantly enriched with MMP13 compared with EPs from cells cultured under normoxic conditions [11]. Collectively, these studies suggest that EPs from NPC cells (in vitro) may function as envelopes that deliver MMP-13 between normoxic and hypoxic cancer cells. Additional validation is needed to develop these findings as several important technical considerations remain unclear such as whether FBS that was used during cell culture was completely devoid of bovine EVs.

Up to 60% of patients with NPC experience chemo- or radio-resistance after primary treatment. In line with previous studies, a variety of mechanisms responsible for this have been proposed including enhanced EMT. Latent membrane protein 1 (LMP1) is a primary oncoprotein encoded by Epstein-Barr virus (EBV) and is a core driver of NPC. Remarkably, LMP1 has been shown to regulate its own transport into CD63^+^/HSP70^+^ EVs via NF-κB activation suggesting that EV secretion may be an important strategy for LMP1 to avoid degradation [12,13]. EV packaging may also be an important mechanism for LMP1 to participate in the intercellular signal exchange and enable its continued role in promoting tumor growth. In fact, CD63^+^/CD81^+^/HSP70^+^ EVs rich with LMP1 from in vitro cultured NPC cells have been shown to confer radio-resistance to recipient NPC cells via activation of P38 MAPK signaling [14]. The relationship between EVs and LMP1 is complex and likely pro-tumorigenic considering the ability of LMP1^+^ EVs to induce cell proliferation, invasion and confer radio-resistance flanked by the apparent nature of LMP1 to induce EV secretion. Of translational relevance, Zuo et al., demonstrated that aspirin reversed EMT by promoting miR-203 expression in NPC cells via repressing LMP1^+^ EP secretion [12]. This is one of the first studies to attempt to counterbalance the relationship between EV and LMP1. Future studies to disrupt the crosstalk between LMP1 and EVs are warranted based on these recent findings.

Though EVs may not be the chief culprit of HNC, they are important mediators of disease progression. A recent in vitro study reported that miR-24-3p is highly enriched in CD63^+^/CD81^+^/TSG101^+^ EVs from the saliva of patients with oral cavity squamous cell carcinoma and oral tongue cancer (HSC-3) cells. Further, it was shown that EVs rich with miR-24-3p stimulated cell proliferation via modulation of Period 1, an important circadian clock gene [15]. While these results must be interpreted with caution based on the crude isolation methodology that was employed and lack of discrimination between tumor-specific and host-derived EPs that undoubtedly co-exist in saliva, they are interesting and warrant validation. The proteomic cargo of CD9^+^ EVs from the same cell line (HSC-3) was examined recently and is characterized by multiple oncogenic proteins, namely EpCAM, EGFR, and HSP90 [16,17]. Based on the traditional and well-established roles of these proteins, in particular HSP90, CD9^+^ EVs from oral cancer cells appear to be equipped to promote tumor growth and metastasis via actively contributing to protein folding, shuttling, and downstream signaling. Considering that EGFR is overexpressed in over 80% of HNCs and has important biological and translational implications, the ability of EGFR rich CD9^+^ EVs to transform oral tongue cancer cells into vimentin-positive, E-cadherin low, mesenchymal phenotypes indicates that they may play an important role in initiating and promoting invasion and metastases [17]. While these results make sense from a biological and clinical perspective, they require validation before their relevance can truly be appreciated. It will be important for future studies to characterize the subpopulations of EVs that are driving these effects.

### 2.2. EVs Regulate Tumor Immunology

HNCs are considered immunologically hot tumors and are heavily infiltrated by lymphocytes, macrophages, and other active immune cells. Despite this, there appears to be a complete failure of the immune response to mount a coordinated and sustained response [18]. As one of the most immunosuppressive malignancies, the integrity of the antitumor immune response is critical for a successful response to therapy. The underlying communication networks linking tumor and immune cells may be governed by EVs, a notion that is supported by studies showing that EVs from HNC cells (in vitro) can reprogram immune cells. Remarkably, host-derived TSG101^+^ EVs from the plasma of patients with active disease (HNC) have been shown to induce significantly greater apoptosis of cytotoxic CD8^+^ T cells, greater inhibition of T cell proliferation or NKG2D expression on NK cells, and enhanced upregulation of suppressor functions in CD4^+^CD39^+^ T regulatory cells compared with EVs from patients with no evidence of disease [19]. In support of this, Wang et al., recently showed that CD63^+^/CD9^+^/Alix^+^/Rab5^+^/HSP70^+^ EVs from oral tongue cancer (CAL-27 and SCC25) cells were specifically internalized by NK cells and promote NK cell proliferation and cytotoxicity by increasing the release of perforin and granzyme M as well as various chemokines and interferons [20]. Further, NF-κB-activating kinase-associated protein 1 was enriched in EVs and appears to be the core driver of enhanced NK cell activity based on in vitro investigations. In a separate study, Maybruck et al., also showed that EVs from the oral cavity (floor of the mouth, Tu167) cells and other previously uncharacterized HNC (SCC0209 and HN60) cells induced a suppressive phenotype in cytotoxic CD8^+^ T cells [21]. Rather than traditional western blotting and similar methods, this study used mass spectrometry to verify that EVs were successfully isolated based on the identification of classical EV markers via in silico analysis. Collectively, these data suggest that EVs from HNCs are involved in modulating the immune response and therefore may serve as reliable, non-invasive indicators of EV-mediated immune dysfunction that correlates with disease activity.

There is an underlying contradiction in the literature between the immune-stimulating and suppressing roles of EVs [22]. In support of an immune-stimulatory role, CD63^+^/CD81^+^ EVs from oral tongue cancer (SCC9, SCC4, and CAL-27) cells cultured under normoxic conditions were shown to stimulate T cell expansion and activity at static concentrations (dose-response titers were not reported) [23]. This study also showed that CD63^+^/CD81^+^ EV stimulating effects could be attenuated by EVs from cells grown under hypoxic conditions and were dependent on HSP70-mediated contact between EVs and T cells. Further, it was suggested that oral tongue cancer EVs mediate the hypoxic evolution of the tumor microenvironment through the transportation of specific miRNAs, namely miR-21. Delivery of miR-21 by CD63+/CD81+ EVs from oral tongue cancer resulted in the downregulation of PTEN, increased expression of PD-L1, and induction of the immunosuppressive activity of myeloid-derived stem cells (MDSCs). Collectively, the presence of MDSCs appears to be crucial for EV miR-21-induced suppression of T-cell cytotoxicity, suggesting the potential of dual inhibition of EVs plus immune checkpoint markers as a novel therapeutic modality for oral tongue cancer. It will be important for future studies to refine these important and elegant findings by elucidating which subpopulations of EVs are responsible for mediating the immune response.

HPV16 infection is primarily associated with tumors of the oropharynx (including the tonsils and base of tongue) and occurs commonly in young patients (<45 years of age). HPV status drives a clinical and molecular dichotomy among patients as those with HPV negative disease tend to have a poorer prognosis whereas patients with HPV positive disease are more likely to respond to initial therapy. This appears to be independent of an intrinsic sensitivity to chemotherapy and/or radiation but rather, may involve the host immune system based on the detection of elevated levels of specific immune effector cells in patients with HPV positive disease [24]. A seminal study reported that HSP70^+^/TSG101^+^ EVs from HPV positive and negative cells and plasma of patients with HNCs differentially reprogrammed recipient immune cells [25]. That is, HSP70^+^/TSG101^+^ EVs from HPV negative oral tongue cancer (PCI-13, PCI-30) cells dampened dendritic cell (DC) maturation whereas EVs from HPV positive oral tongue cancer (UMSCC2, UMSCC47, and SCC90) cells promoted maturation and maintenance of DC functions in anti-tumor immune responses, consistent with improved outcomes for HPV positive patients. A comprehensive proteomic analysis of the membrane components of HSP70^+^/TSG101^+^ EVs from HPV positive and negative cells suggested that this may be attributed to differential enrichment of biologically active membrane-associated proteins that may preferentially promote immune interactions among HPV cancers, or protect tumor cells from adverse immune or drug effects for HPV negative disease. Indeed, Ludwig et al., reported that HPV^+^ EVs were enriched with immune cell-related effectors, such as CD47 and CD276, while HPV^-^ EVs were enriched with tumor-protective and growth-promoting antigens, such as MUC-1 and HLA-DA [19]. In the context of these recent studies, EVs appear to be important mediators of tumor immunity that are governed by HPV status. Further in vivo work and a better appreciation of the specific EV subpopulations that act as specific antigen-presenting vehicles are now needed to progress this work further.

Although immune checkpoint inhibitors have revolutionized therapy for some cancers, response rates in HNCs have been disappointing [26]. While previous studies have hinted that this may be related to an abundance of EVs with immunosuppressive properties [19], others have explored the possibility that EVs may express immune checkpoint markers that facilitate immune escape. A recent study showed that PD-L1^+^ EVs downregulated CD69 expression on activated T cells, an effect that was almost completely reversed by pre-incubating T cells with anti–PD-1 [27]. Further, PD-L1^+^ EVs from plasma of patients with HNC but not circulating, free PD-L1 was shown to correlate with clinico-pathological parameters such as the evidence of advancing disease, the high tumor stage and the lymph node involvement. The ability of EVs to transport functional PD-L1 and subsequently modulate immune cells emphasizes their importance in antitumor immunity.

### 2.3. EVs Promote Angiogenesis

Angiogenesis is the formation of new blood vessels that facilitate tumor growth, progression and metastasis by availing oxygen, nutrients, or cell metastatic conduits. Various studies have shown that EV uptake induces upregulation of angiogenesis-related molecules and results in enhanced endothelial cell proliferation, migration, sprouting and maturation [28,29,30]. Recent work has suggested that enhanced angiogenesis may be mediated by selective packaging of miR-142-3p into small EVs (sEVs) [31]. This study showed that miR-142-3p could induce both angiogenesis and vascular density in vitro and in vivo. Other studies have shown that a variety of other miRNA species are packaged into EVs and may also regulate angiogenesis in HNCs including miR-23a [32], miR-17-5p [33], miR-9 [34]. However, the explicit targets and mechanisms by which these miRNAs modulate angiogenesis are yet to be completely elucidated. Currently, it is unknown whether certain miRNAs act alone or in concert with other molecular cargo.

EV proteins can also directly activate angiogenesis associated pathways. A recent study reported that oral tongue (OSC19 and SCC61) or pharyngeal cancer (Detroit 562) cell-derived sEVs can induce Ephrin-B reverse signaling in endothelial cells and promote angiogenesis via Ephrin type B receptor 2 (EPHB2) [35]. EPHB2 carried on sEV was shown to bind to receptors on endothelial cells, activate STAT3 and VEGF and increase transcription of angiogenesis-related genes. Other studies have shown that CD63^+^/CD9^+^/Flotillin-1^+^ EVs from NPC (CNE2, CNE1, 5-8F, 6-18B) cells were enriched with a key glycolysis-regulatory enzyme, 6-Phosphofructo 2-kinase/fructose 2, 6-bisphosphatase 3, which promotes angiogenesis amongst other tumor permissive activities [36]. More recently, CD9^+^/TSG101^+^ EVs from HPV positive oral tongue cancer (UMSCC47) cells were shown to promote angiogenesis directly by interaction with endothelial cells and indirectly via the upregulation of adenosine and the A_2B_R-mediated pathway in macrophages [37]. This area of EV research is provocative but needs more time to develop. An appreciation for the specific differences between EV subtypes arising from specific cells and precise mechanisms governing EV driven angiogenesis are far from being fully appreciated. There is much heterogeneity in clinically derived EVs that currently prohibits that translation of these important yet largely in vitro findings.

### 2.4. EVs Remodel the Tumor Microenvironment

The tumor microenvironment is composed of: tumor cells; cancer-associated fibroblasts (CAFs); tumor-associated macrophages (TAMs); immune cells; stromal cells; vascular cells; extracellular matrix; and a large repertoire of secretory products, all of which play a vital role in tumorigenesis and tumor development [38]. CAFs are an abundant cell type in the tumor microenvironment and are associated with poor prognosis in HNCs [39]. As such it is not surprising that there has been extensive research exploring CAF transformation and activation by HNC EVs [40]. EVs are a formidable tool that tumor cells use to re-educate adjacent fibroblasts into CAFs. For example, CD63^+^/CD81^+^/TSG101^+^ EVs from the salivary adenoid cystic carcinoma cell line (SACC-83) were readily internalized by normal, human umbilical vein endothelial cells (HUVECs) and periodontal ligament fibroblasts (HPLFs) and drove a malignant phenotype in recipient cells [41,42]. It was suggested that this occurred via promotion of pro-inflammatory cytokine and nerve growth factor secretion but more studies are needed to confirm this. Another example was recently reported where CD63^+^/CD81^+^/TSG101^+^ EVs enriched LMP1 from NPC cells transformed normal fibroblasts into CAFs [43]. This study also showed that LMP1^+^ EVs induced autophagy and drove metabolic switching in CAFs that ultimately promoted the proliferation, migration, and radiation resistance of NPC cells. However, once again further work is needed to provide compelling evidence that these effects are directly associated with EVs rather than external cargo.

Although TAMs classically harbor two phenotypes, anti-tumoral M1 and pro-tumoral M2, a high density of TAMs in HNCs correlates with a poor prognosis [44]. This suggests that there may be tumoral-driven, real-time programming of TAMs that occurs on an “as needed” basis. Indeed, EPs from oral tongue cancer cells (CAL-27 and SCC9) were shown to promote M2 polarization (in general) [45]. In addition, EPs from transformed hypopharyngeal cells (FaDu) were also shown to drive M2 polarization via miR-21 suppression of PDCD4 and IL12A [46]. A more recent study using EVs from the floor of the mouth (SCC104) and oral tongue (SCC90, SCC47, SAS, CAL-27, and CAL33) cells revealed that HPV status can dichotomize TAM polarization [47]. Specifically, CD9^+^/CD63^+^/TSG101^+^ EVs from HPV positive cells (SCC47, SCC90, and SCC104) induced M1 polarization whereas EVs from HPV negative cells (SAS, CAL-27, and CAL33) induced M2 polarization. Although more work is needed, it is tempting to speculate that EV driven M1 polarization in patients with HPV positive disease may be important in mediating favorable clinical outcomes. Along these lines, CD9^+^/CD63^+^/TSG101^+^ EVs from HPV positive cells have also been shown to promote radio-sensitivity among HPV negative cells and that miR-9 was an important factor driving this [47]. An earlier study claimed to have shown that EVs from HPV negative cells (SCC25 and CAL-27) drove the opposite effect (induction of M1 polarization) but little controversy exists based on the fact that EVs were not isolated [48]. Instead, crude culture media was concentrated and used in the place of EVs. There is little doubt that concentrated cell culture media will elicit different effects when compared with purified EVs.

Interestingly, macrophages, which canonically phagocytize cell debris, can also favor the dissemination of EVs. Macrophages that have phagocytosed EVs appear to be highly invasive and can release membrane blebs containing EVs to stromal cells. As such, it is not surprising that EVs from macrophages can reciprocally educate tumor cells, albeit in a secondhand manner. In support of this, Alix^+^/TSG101^+^ EVs from unstimulated macrophages were shown to promote the migration of laryngeal cancer cells (BICR18) as well as boost an immunosuppressive state by inducing the expression of PD-L1 [49]. Additional in vivo studies are now needed to verify these novel findings and fully appreciate the clinical and potential therapeutic relevance.

### 2.5. EVs Regulate Drug Resistance

The leading cause of treatment failure among patients with HNCs is drug resistance, especially acquired drug resistance. The mechanisms governing drug resistance include but are not limited to drug inactivation, drug target alteration, epigenetic modifications, drug efflux, DNA damage repair, cell death inhibition, and induced EMT. A few studies have now shown that intracellular cisplatin concentration in oral cancer cells dramatically increases after inhibiting the secretion of EVs [50,51]. In addition, drug-resistant cells appear to expel cisplatin more rapidly via enhanced sEV packaging and secretion when compared with non-resistant cells. It was proposed that the underlying mechanism of cisplatin resistance may be mediated via miR-21 targeting of phosphatase tensin homolog (PTEN) and programmed cell death factor 4 (PDCD4) [51]. More recently, EP transmission of miR-30a was shown to correspond with the development of cisplatin resistance via the upregulation of Beclin1, an autophagy-related gene [52]. Adding to this, another recent study revealed that transmission of EP miR-155 was associated with cisplatin-resistance [53]. However, before the precise EV cargo that are involved in cisplatin resistance can be defined, better characterization of the EVs and their subpopulations are required. Further, additional in vivo work is required along with studies addressing the impact of combined chemotherapy and specific miRNA inhibitors.

Taking an alternative hypothesis, Chen et al., suggested that oral cancer stem cell (CSC)-derived EPs may play a role in the development of cisplatin resistance [54]. Crude EPs from CSCs were shown to transport oncogenic cargo and promote cisplatin resistance, self-renewal, and CAF transformation. Although these pro-tumor features suggest that CSCs utilize targeted signaling networks via EPs, the importance of these data will remain unclear until the precise subpopulations EVs orchestrating these effects are resolved.

As high levels of EGFR strongly correlate with decreased survival, targeted therapies against this marker are a hot topic [55]. Currently, resistance to anti-EGFR therapies is a major problem and much work is underway to understand and overcome this. Cetuximab is a chimeric IgG1 monoclonal antibody that competitively inhibits ligand binding to EGFR, fostering EGFR internalization and altered EGFR dependent signaling. As mentioned, in vitro analyses have revealed that cetuximab can promote the secretion of EGFR CD9^+^ EVs by oral tongue cancer (HSC3) cells but fails to inhibit EGF-driven secretion of EVs [17]. Of biological relevance, cetuximab inhibited the ability of CD9^+^ EVs to initiate and promote EMT but was unable to block EGF driven mesenchymal progression of transformed cells, apparently due to selective packaging of the drug into EGFR^+^ EVs. Whether this is a physiologically relevant targeted drug resistance mechanism that is responsible for refractory, resistant cancer remains to be established. Like BRAF resistance in melanoma, other downstream signaling molecules may be involved in resistance including mutations in PI3K, PTEN, or Ras. In future studies, it will be important to consider the plethora of alternative escape mechanisms already proposed including but not limited to additional activation of HER3 signaling, compensatory activation of MET, overexpression, and hyper-activation of AXL, aberrant cell cycling via p53 mutations [55]. Considering the poor overall survival rates of patients with advanced HNCs, further work is needed in this area.

### 2.6. EVs Regulate Radio-Sensitivity

For the treatment of locally advanced disease, radiation therapy is employed as an adjunct to surgery or is administered concurrently with chemotherapy. However, radio-resistance leading to local recurrence and metastasis are common complications. There is overwhelming evidence that EVs are important mediators of radio-resistance. Towards this, a range of studies have shown that that the protein content of sEVs from irradiated cells (hypopharyngeal FaDu cells and oral tongue UM-SCC6 cells) are markedly different compared with non-irradiated cells and are characterized by an over-representation of proteins involved in transcription, translation, protein turnover, cell division and cell signaling [56,57,58]. This seems counterintuitive at first as one would expect suppression of transcription, translation, and general cell functionality in response to radiation. However, it appears that EV packaging of proteins involved in these crucial mechanisms may reflect a dynamic adaptation of cells to stress-induced conditions via removal of excessive components. That is, EV cargo from irradiated cells suggests that there may be temporary and transient suppression of transcription and translation which may explain radio0resistance. In further support of this, another study using hypopharyngeal (FaDu) cells showed that sEVs from irradiated cells resulted in increased DNA double-strand break repair and stronger pro-survival effects compared with EVs from non-irradiated cells [59]. This study also showed that radiation drove increased sEV release and uptake by HNC cells. However, as the number of sEVs did not correlate with the pro-survival phenotype it was suggested that the sEV cargo, namely RNA, may be primarily responsible for inducing the repair process in recipient cells. Considering the likely functional role for EVs in the response of tumor cells to radiation and their ability to reach a broad “community” of nearby and distant cells, the therapeutic potential of targeting EVs or their specialized cargo is worthy of further consideration.

## 3. EVs as a Potential Biomarker of HNCs

**Figure 4:**
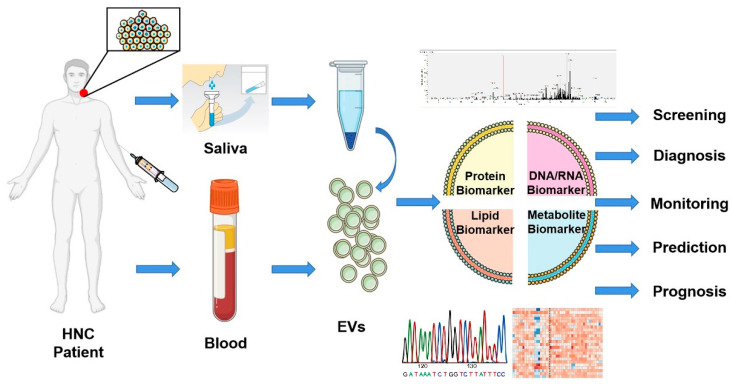
Biomarker potential of extracellular vesicles. Liquid biopsies based on EVs offer a promising non-invasive method for screening, diagnosis, monitoring, prediction, and prognosis of head and neck cancer patients. EVs isolated from saliva or blood can be analyzed through multi-omics studies and offer a tremendous opportunity to improve the clinical management of patients. This figure was created with Smart Servier (https://smart.servier.com) and BioRender.com.

Biomarkers for monitoring HNC disease burden, therapeutic response, or metastatic potential are lacking. Therefore, a pressing task is to develop a sensitive and specific yet simple, economical, and robust detection method (Figure 4). EVs are secreted by all cells including tumor cells and resemble, to some degree, the molecular cargo of their parental cells [60]. Although an appealing non-invasive source of EVs are biofluids, it is crucial to acknowledge that plasma, saliva, and other body fluids are comprised of host-derived EVs and provide an aggregated representation of the tumor and host [61].

Besides EVs, circulating tumor cells (CTC) and cfDNA are promising alternative liquid biopsies. Nonetheless, EVs, compared with CTC and cfDNA, have unique advantages: (1) EVs are pro-actively released by tumor and other cells rather than being passively released as a consequence of apoptosis; (2) EVs and their contents are comparatively stable because of the protective lipid bilayer; (3) EVs are small-sized and highly permeable to the extracellular matrix [62]; (4) EVs are comprised of a range of molecular cargo which is favorable for discovery of tumor-specific biomarkers [63]. Multi-omics and next-generation sequencing techniques continue to evolve and facilitate a deeper dive into the nature and contents of EV cargo. Although we are cautious with our interpretation of the current translational EV literature, their unique advantages prompt consideration.

### 3.1. Biomarker Potential of EV Levels

EVs are highly heterogeneous and are secreted by many cells including non-tumor, immune, stromal, and others [64,65,66]. However, this may not be such a bad thing because the heterogeneity of EVs identifies with tumor heterogeneity and tumor-induced host responses, which suggests that EVs may offer a holistic perspective with a collectively stronger biomarker potential [67]. Supporting this, a direct comparison of salivary and plasma medium/large EVs (m/lEVs) from patients with oral cancer revealed that the level of saliva m/lEVs was elevated concomitant with the level of plasma m/lEVs and was associated with lymph node metastasis and higher clinical stage [68]. These results have been corroborated by earlier studies showing that saliva EVs from oral cancer patients are greater in size, more irregular in their morphology, and aggregate more readily compared with EVs from controls [69,70]. Beyond this, a recent study demonstrated that CD63^+^/CD9^+^ EVs from the saliva of patients with oral cancers have unique and discriminatory infrared signatures compared with healthy controls [71]. Overall, these results are encouraging even though the EVs in saliva are an admixture of nanoparticles that are released by both tumor and non-tumor cells in the oral cavity.

Only one study has compared EV levels from the plasma of patients with oral cancer before and after surgery and showed that certain subpopulations of EVs were decreased (CD63^+^) or increased (Calveolin-1^+^) [72]. However statistical significance was not reached likely due to the small study size (*n* = 10). Another study evaluated the predictive value of plasma CD63^+^/CD9^+^/CD81^+^ EVs to distinguish patients who benefited from therapy from those who did not [73]. Besides observing that plasma EV protein levels in HNC patients were higher at baseline compared with healthy controls, the authors noted that plasma EV protein levels increased during therapy only in patients who experienced a recurrence. In contrast, plasma EV levels decreased relative to pre-therapy levels in cases who completely responded to treatment. Another important observation was that the amount of tumor-specific EVs (enriched using tumor-specific markers anti-EGFR, anti-MAGEA3, anti-EpCAM, and anti-CSPG-4) varied among different patients. Finally, this study also showed that CD3^+^, CD3^-^/PD-L1^+^, and CD3^+^/15s^+^ (T regulatory cell-derived) EVs increased after treatment (with ipilimumab) in patients whose disease recurred whereas corresponding EV populations stabilized in patients who remained disease-free. This is one of the first studies in the HNC field that has investigated the levels of subpopulations of EVs and we hope that others build upon these encouraging findings.

### 3.2. Biomarker Potential of EV DNA Cargo

EV DNA is a controversial topic. While many argue that the biosynthesis of EVs is inconsistent with DNA packaging and that EV DNA cargo is “non-specific” [74], others have forged ahead with EV DNA analyses [75]. Genomic DNA including single-stranded and double-stranded, mitochondrial DNA (mtDNA), as well as some specific oncogenic amplifications have been reported as EV cargo [76,77,78,79]. A seminal study of paired tumor tissues and CD63^+^/TSG101^+^ EVs from the serum of patients with pancreatic cancer reported that EV DNA covered the entire genome, and carried mutations that were identical to the parental tumors [80]. Further, EV DNA appears to be actively transported and not only affects corresponding mRNA and protein levels that subsequently affect cell function [81,82] but also reflects the mutational status of parental tumor cells without the need for direct tumor sampling [83]. Recently, EGFR mutations within EP DNA showed a sensitivity of 100% and specificity of 96.55% in comparison with the detection of tumor tissues in lung adenocarcinoma [84]. This led to the development of an EV-based detection method called ExoDx EGFR that uses qPCR to interrogate EGFR mutations in EV RNA/DNA and cfDNA with high sensitivity and specificity [85,86,87]. This is relevant for HNCs which are frequently characterized by EGFR mutations. Future studies may assess whether early and non-invasive detection of EV-EGFR mutations by ExoDx EGFR may inform clinical management through induction of targeted therapy (cetuximab, a monoclonal antibody directed against EGFR) for advanced HNCs [88].

Considering the importance of certain viruses in tumorigenesis, some have turned their attention to explore the capacity of EVs for viral DNA packaging. EVs have demonstrated the capacity to convey HPV transmission in cervical cancer but their role in HPV-driven oropharyngeal cancers has not been explored [89]. Nguyen et al. were the first to report that CD63^+^/CD9^+^/TSG101^+^ EVs derived from the plasma of HPV positive base of tongue cancer patients contained molecular components of HPV including HPV DNA. However, compared with cell-free HPV DNA, EV HPV DNA had a lower sensitivity [90]. Another recent study examined HPV DNA in CD63^+^/HSP90^+^/TSG101^+^ EVs from the saliva of patients with oropharyngeal cancer using a novel acoustofluidic platform. While this method was rapid and insensitive to salivary viscosity, it is highly technical and achieved only 80% concordance with the gold standard (immunohistochemistry for p16) [91]. This is an exciting area of research that is clearly in its infancy.

While studies continue to show that EVs have good selectivity for tumor DNA [80,92], we acknowledge that others disagree and that there are major gaps in knowledge. A recent study suggested that the loading of DNA by EVs is a random, non-selective process [74]. This study showed that DNA fragments within EVs from different cell lines had no obvious genomic selectivity. Further, it was shown that dsDNA was non-vesicular and was secreted in autophagy and multivesicular-endosomes-dependent manner. Beyond this, there appear to be different “loading patterns” among different types of vesicles [93]. For example, relatively large EVs whose diameters are greater than 1 μm were shown to contain most of the free DNA fragments in plasma whereas small EVs, such as EVs contained negligible amounts of DNA fragments [92]. We speculate that different conclusions may be related to the types of samples used, the pre-processing methods employed, and differing types of EVs studied. All of these are crucial factors that underscore the need for further research.

Overall, there is very little research focusing on EV DNA in HNCs and several important questions remain. Are critical oncogenic mutations concentrated in EVs and do these robustly reflect the mutational status of parental tumor cells? Does EV DNA precede cell-free DNA as a clinical biomarker? Can salivary EVs be used for HPV or EBV detection with higher sensitivity than current gold standard testing methods? Are epigenetic alterations of EV DNA detectable and useful as novel biomarkers?

### 3.3. Biomarker Potential of EV RNA Cargo

In recent years, EVs have been the focus of intense research owing to their capacity to transfer RNA molecules, especially miRNAs, lncRNAs, and circRNAs to recipient cells. miRNAs are a type of single-stranded, non-coding short RNA molecules of approximately 20 nucleotides in length, which play important roles in the modulation of the various physiological and pathological processes by forming the RNA-induced silencing complex to degrade mRNA of targeted genes. The biomarker potential of miRNAs associated with EVs has been explored in various cancer types including HNC [94,95]. EP-associated miR-21 has been shown to mirror the status of tumor miR-21 expression in patients with oral cancer underscoring its biomarker potential [96]. This was supported by an earlier study that showed combined detection of plasma EP associated miR-21 and HOTAIR (a long non-coding RNA) could distinguish malignant laryngeal cancer from the benign disease [97]. This study also showed that there were significant differences in circulating miR-21 and HOTAIR associated with EPs between cases with different levels of disease burden and that this correlated with clinical parameters. Beyond miR-21, EP associated miR-941 was also shown to be highly expressed in the plasma of patients with laryngeal cancer compared with healthy controls [98]. A few recent studies have suggested that there are discrete miRNA signatures associated with plasma EVs that can discriminate between patients with the premalignant disease (oral lichen planus) and healthy controls [99] or patients with papillary thyroid cancer and healthy controls or benign thyroid nodules [100]. Similarly, a more recent study showed that miRNA associated with EPs from the serum of patients with nasopharyngeal cancer matched tumor miRNA more reliably than circulating free miRNA [101]. Most studies discussed in this section used crude isolation methods and lacked robust validation for bona fide EVs. While this is noteworthy, it is not necessarily a flaw considering the collective goal of biomarker discovery. Overall, these preliminary studies are important and should guide future efforts.

Circular RNAs (circRNAs) are a unique class of RNAs and are comprised of large non-coding RNAs that are produced by non-canonical splicing. CircRNAs have important roles in gene regulation via their capacity to bind to and inhibit miRNAs. Further, they are thought to have greater biomarker potential than their linear RNA (miRNA) counterparts due to enhanced stability attributed to their circular structure. A comparison of paired laryngeal cancer and adjacent normal tissues revealed that circRASSF2 was upregulated in laryngeal cancer tissues [102]. The authors then showed that circRASSF2 was also upregulated in EPs. Another circRNA species, circRNA_0000199 was recently shown to have prognostic potential whereby upregulated expression correlated with betel nut consumption, tumor size, lymph node involvement, and TNM stage of oral cancer patients [103]. Specifically, patients with high levels of circRNA_0000199 in CD63^+^/TSG101^+^ EVs EPs had high tumor recurrence and mortality rates underscoring the prognostic potential. These conclusions are relatively new and further studies are needed to verify these findings.

Besides plasma, the miRNA content of saliva has also been investigated. An early study compared HNC and normal human oral epithelial cells (in vitro) and revealed that differential expression of miRNA associated with EPs was consistent with patterns observed from analyses of saliva EPs from HNC patients and healthy controls [104]. Building on this, another study revealed that some miRNAs were exclusive (miR-302b-3p and miR-517b-3p) to CD63^+^/CD9^+^/Alix^+^/TSG101^+^ EVs from the saliva of patients with oral cancer compared with controls whereas others were up-regulated (miR-512-3p and miR-412-3p) [105]. A more recent study showed that miR-24-3p was one of the most significantly upregulated miRNAs associated with CD9^+^ EVs from the saliva of patients with oral cancer [15]. This is consistent with the proposed function of miR-24-3p in oral cancer cells which is to produce a “tumor-friendly” environment that promotes cell proliferation by regulating the expression of cell cycle-related genes. Although ROC analysis revealed that EP miR-24-3p could significantly distinguish oral cancer patients from normal controls with reasonable accuracy (64.4% sensitivity and 80% specificity), more evidence is needed before the clinical utility of this and any other miRNA biomarker can be realized.

EP associated Epstein-Barr Virus BART13-3p miRNA has been shown to distinguish nasopharyngeal cancer cases from patients with other HNC subtypes and patients with asymptomatic EBV infection [106]. This is the first data to suggest that EP packaged EBV miRNA could be a promising, NPC-selective biomarker as a screening strategy to identify NPC patients. As already mentioned, the HPV status of oropharyngeal carcinoma is strongly related to prognosis. Considering this, CD63^+^/CD9^+^/TSG101^+^ EV miRNAs from HPV positive and HPV negative cells were compared, and discrete signatures of miRNAs that are associated with HPV status were revealed [107]. This work paves the way towards the measurement of miRNAs as a useful surrogate for HPV status. Whether a similar approach can reveal miRNAs to accurately stratify patients with and without lymph node involvement or those who will or will not experience disease recurrence is yet to be explored.

### 3.4. Biomarker Potential of EV Protein Cargo

It is well established that the composition of EVs changes during disease onset and progression as well as in response to chemotherapy and/or radiation therapy. Considering that EVs serve as a carrier for signal molecules, of which proteins are dominant, several studies have used proteomics to investigate EV cargo from cell lines [57,58,59]. However, we have limited this section to studies that have used clinical samples in an attempt to focus on the translational potential of EVs.

Like other cargo types, proteins appear to be preferentially enriched in EVs, and targeted studies that are hypothesis-driven have led to significant advances in the field. Beyond potential biomarkers including EGFR and PD-L1, CD44 has emerged as a useful candidate in HNC. The CD44v3 variant is particularly interesting as its overexpression in HNCs is associated with increased proliferation, migration, and enhanced metastatic potential as well as a poor prognosis [108,109,110]. Based on this, a recent study showed that CD63^+^/CD9^+^/CD81^+^/TSG101^+^ EVs enriched with CD44v3 from the plasma of patients with HNCs was associated with disease stage, nodal metastasis, and immune dysfunction [111]. Other targeted studies have shown that elevated levels of certain proteins (Lysyl oxidase-like 2 and Cyclophilin A) are associated with EPs in the plasma of patients with HNCs and correlate with disease status [112,113]. Importantly, it was shown that when Cyclophilin A levels in CD63^+^/TSG101^+^/HSP70^+^ EVs were combined with EBV viral capsid antigen (VCA-IgA) levels, they acted synergistically to provide a combinatorial biomarker with improved accuracy of NPC diagnosis. Although EBV DNA is an outstanding tool for early diagnosis [114], this study suggests that additional markers for NPC may further improve the management of this disease.

Beyond single protein analyses, larger studies have also been performed. An interesting study used Cholerae Toxin chain B (CTB) and Annexin V (AV) to enrich subpopulations of EPs from pools of plasma-based on the theory that EVs are enriched with specific proteins that specifically bind to these (e.g., phospholipids such as GM1 ganglioside and phosphatidylserine) [115]. Although the presence of EPs was not validated, an antibody array-based analysis detected 370 proteins (out of a possible 656) in plasma from patients with HNCs. Of these, 119 proteins were specific to patients who failed to respond to chemo-radiation therapy while 38 were exclusive to complete responders. EPs from patients who failed to respond were enriched with several proteins that have been reported previously including FAS, RET, STAT5, TNFRSF1B, WNT1, ABCB1, CASP5, CCND1, FGF1, ABL1, BCL2L1, PRIM1, CD4, HSP90AA1, and HSP90AB1. In contrast, a discrete set of proteins were enriched in EPs from patients who achieved a complete response including BAX, CASP3, HDAC1, NGFR, TNFSF11, TP73, BRCA2, EGFR, IKBKB, STAT1, SNAI1, BAG1, and TNFRSF10B. Larger studies are now needed to validate these findings to appreciate their translational potential.

Owing to continual advances in mass spectrometry, untargeted proteomics approaches are beginning to reveal even more about the clinical capacity of EVs. The first study to use label-free quantification to investigate the role of EPs from the saliva of patients with HNCs focused on the effect of the immune and inflammatory response on tumor growth [116]. Three comparative analyses were performed: (1) whole saliva from patients with oral cancer versus controls; (2) whole saliva from patients with oral cancer who exhibited lesions versus those who did not; (3) saliva EPs from patients with oral cancer versus controls. Despite using relatively crude isolation and minimal validation methods as well as studying a heterogeneous cohort of HNCs, several hundred proteins were identified. Of these, 8 proteins were differentially expressed between oral cancer cases and controls, 18 proteins were private to healthy controls and 4 proteins were private to the oral cancer group. It is unclear whether any proteins identified in the EPs were also identified in the whole saliva. While assumptions and interpretations were made based on in silico analyses of these findings, more rigorous EV isolation and validation are required before meaningful hypothesizes can be drawn. More recently, label-free quantification was used to identify 415 proteins in CD63^+^/CD9^+^/TSG101^+^ EVs from the plasma of patients with oral cancer [117]. Four proteins (PF4V1, ApoA1, CXCL7, and F13A1) were chosen for further validation according to the bioinformatics analysis, commercially available antibodies and ELISA kits, and evidence from previous research. Although a few potentially significant relationships with clinicopathological factors were identified, no connection with overall survival was reported. This is an exciting study and the first to explore plasma EVs in HNCs but suffered from an important limitation. EVs were isolated using a crude precipitation-based approach that is notorious for co-isolating “sticky” high abundant serum proteins. As a result, a large proportion of low abundant proteins may have been masked and therefore missed. Future studies to validate the importance of EV proteins that were private to cases with lymph node metastases may be quite revealing. In addition, similar studies focusing on subpopulations of EVs will no doubt be helpful.

## 4. EVs and Treatment of HNCs

**Figure 5:**
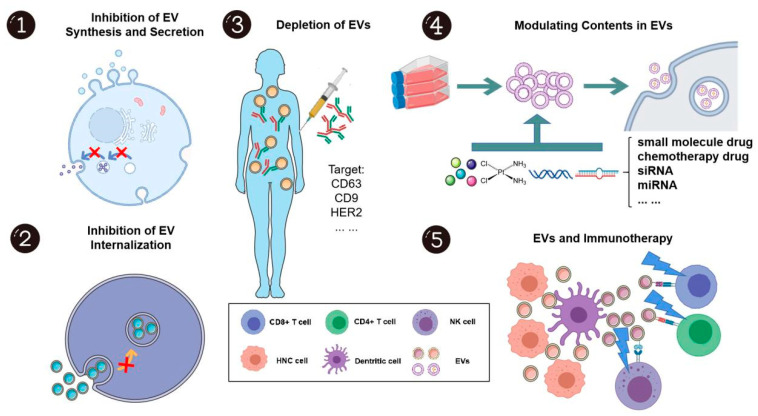
The potential applications of extracellular vesicles in head and neck cancer treatment. EVs show great potential as both treatment targets and drug carriers. As EVs can promote head and neck cancer development and progression, inhibition of synthesis, secretion, internalization, and even the depletion of EVs, to some extent, may elicit anti-cancer effects. Further, EVs are unique in that they can encapsulate drugs or bioactive molecules as drug delivery systems due to their high bioavailability, biocompatibility, and positive targeting capacity. EVs may also disrupt the immunosuppressive environment and be novel “adjuvants” for immunotherapy. This figure was created with Smart Servier (https://smart.servier.com) and BioRender.com.

As EVs play a significant role in HNC carcinogenesis, development, and progression, many researchers have considered their capacity as therapeutic targets and novel drug delivery systems (Figure 5).

### 4.1. EV as a Direct Target: Inhibition of EV Synthesis and Secretion

EVs are formed during multi-vesicular body biogenesis, a process that is mediated by endosomal sorting complexes required for transport (ESCRT)-dependent and ESCRT-independent pathways. Thus far, various compounds have been shown to target this process. The ESCRT-independent pathway is mediated by neutral sphingomyelinases, which converts sphingomyelin to ceramide. It is known that neutral sphingomyelinase is crucial in endosomal sorting and EV biogenesis. GW4869, a neutral sphingomyelinase inhibitor, has been shown to prevent the formation of intraluminal vesicles and thus block the production of EVs [118]. In the context of HNCs, infiltration of nerves into the tumor parenchyma contributes to tumor progression, such that patients with densely nerve innervated tumors tend to have worse outcomes [119,120]. Madeo et al. firstly showed that GW4867 significantly reduced the release of CD9^+^/CD81^+^ EVs from HNC tumors (grown in vivo) and in parallel, observed a reduction in the formation of tumoral nerves that were comprised of unorganized β-III tubulin (neuron-specific) positive fibers [121]. Such findings suggested that HNC cells release a subpopulation CD9^+^/CD81^+^ EVs that mediate sensory fiber axonogenesis in cancer. Building on this, Amit et al. reported that *TP53* mutated HNCs induce sensory nerve differentiation into adrenergic fiber via miR-34a, miR-21, and miR-324 packaged in CD63^+^ EVs [122]. It was also demonstrated that HNC growth could be inhibited by sensory denervation or blockade of adrenergic receptors by carvedilol (a drug commonly used to treat high blood pressure). On the other hand, the development of new adrenergic nerves was prevented by the ablation of sensory nerves. Taken together, inhibition of EV secretion might decrease sensory fiber formation and possibly their differentiation into adrenergic fibers, which may subsequently decrease tumor growth. In line with the emerging trend of targeting neural regulation in the management of cancer, denervation and targeted suppression of adrenergic activity within tumor infiltration by suppression of EV release may represent a new way for the treatment of HNCs. However, much work is needed to rationalize this novel hypothesis.

There are currently numerous limitations associated with targeting the biogenesis and release of EVs. Firstly, the mechanism of EV biogenesis and secretion has not been completely elucidated. However, it is hoped that this may change with emerging technologies. An example of this was proposed by Lu et al., who developed a novel CRISPR screening platform using artificially barcoded miRNAs (bEXOmiRs) to identify genes involved in EV biology [123]. Additional destructive technologies like this are now required to continue advancing this field. Secondly, as already stated EVs are not only secreted by cancer cells but also by normal (non-pathological) cells and are involved in a diverse repertoire of important physiological functions. For example, EVs from tumor cells (in vitro) have been shown to promote angiogenesis, tumor cell invasion, and immune escape. In sharp contrast, EVs have also been shown to suppress tumor progression via antigen presentation to immune cells. Moreover, dendritic cell-derived EVs carry major histocompatibility complex class I and class II/peptide complexes and can prime other immune cells and activate an immune response [124,125]. Nevertheless, targeting tumor EV release or blocking the ability of nerves to respond to EVs may be of therapeutic value for HNCs. The diversity of EV functionality may be related to the use of heterogeneous EVs composed of subpopulations. This is an area of intense investigation in the EV field and at this time presents more questions than answers related to standardization, study design, and the need for new, disruptive technologies.

### 4.2. Inhibition of EV Internalization

Cells take up EVs by a variety of endocytic pathways, including clathrin-dependent and independent pathways, which may depend on the protein and glycoprotein expressed on EV and target cells. It is likely that EVs may gain entry into a cell via more than one route and that this may be dependent on their subpopulation. Although numerous chemicals and peptides have been shown to block the entry of EVs via different pathways, there is still no in vivo data available about the effects of inhibition of EV internalization. Importantly, there is a lack of understanding of specific mechanisms regarding the key steps in EV trafficking and target definition in HNCs. The Erbitux in First-Line Treatment of Recurrent or Metastatic Head and Neck Cancer Phase III trial reported a significant increase in median overall survival (3.3 months) when cetuximab was added to chemotherapy for untreated recurrent or metastatic HNCs [126]. However, this improved survival was not accountable by the expression of EGFR in the cancer tissues nor any other apparent predictive biomarkers for EGFR, such as *K-RAS,* copy-number-variations, or loss of PTEN. A provocative alternative explanation maybe EVs. Based on a recent report showing that the internalization and pro-mesenchymal effects of oral tongue cancer (HSC3) cell-derived EVs could be blocked by cetuximab, it would be interesting to investigate the relationship between EGFR-rich EVs and survival [17]. Another novel avenue of investigation may include the study of other agents that target EV internalization with higher efficiency. This concept is supported by an earlier study that used heparin to suppress the internalization of CD9^+^/CD63^+^/Rab5^+^/Annexin II^+^ EVs from oral tongue cancer (OSC3 and OSC4) cells via masking heparin sulfate proteoglycans on cancer cell surfaces [127].

### 4.3. Modulating Contents in EVs

The ability of EVs to carry and transport biomolecules from donor to recipient cells as well as cross the blood-brain barrier makes them ideal candidates for drug delivery carriers. Methods to load EVs with cargo have been extensively studied and reviewed elsewhere [128]. In general, these methods include direct incubation with EVs, passive loading by incubation with donor cells, electroporation, sonication, freeze/thaw method, extrusion, chemical conjugation, and formation of nanoparticle-drug complexes. The role of EVs in drug delivery, besides tissue targeting, is to increase the solubility, bioavailability, and stability of therapeutic molecules. Natural compounds such as green tea [129], natural dietary polyphenols [130], and resveratrol [131] are promising companion chemoprevention agents for HNCs. Specifically, green tea has been shown to reduce oral premalignant lesions; leuteolin has been shown to induce apoptosis and inhibit tumor growth in vivo; resveratrol has been shown to induce apoptosis in vitro. Albeit, all three were limited in efficacy due to their poor bioavailability [132,133,134]. EV encapsulation may potentially increase the bioavailability of these and other unappreciated natural compounds preferentially to HNCs.

In line with various preclinical studies on EV-based delivery of natural compounds, efforts are also seen via the clinical trials of oral administration of grape EVs on the prevention of HNC progression (clinical trial: NCT01668849). Unfortunately, results have yet to be published but this is an interesting area of research worth noting. In further support of this, given the initial success of the novel botanical drug, APG-157 which is rich with curcumin, EV-based delivery of a combination of multiple molecules acting synergistically derived from, for example, traditional Chinese herbs, may warrant further investigations.

A recent study showed that crude preparations of EPs from bone marrow mesenchymal stem cells transfected with miR-185 were able to modulate inflammation and promote apoptosis in mice with the oral potentially malignant disorder (OPMD) [135]. Overall, this study reported reduced dysplasia in OPMD and ascribed this to EPs rich with miR-185. However, these results must be interpreted with caution for several reasons: (1) a new EV isolation method was used (Genexosome Technologies) and very few details were provided which is why we refer to the particles as EPs; (2) standard validation was performed but lacked the inclusion of negative markers so the purity of the particles is unclear; (3) dose-response data were not presented. Overall, the larger concern is that the other contents in the crude EP preparations were not considered and may also be responsible for the functional attributes described. Despite these limitations, this study is the first of its kind and is important. The fundamental mechanisms orchestrating miRNA signaling in target cells are largely unknown and warrant further investigations. Further, systemically screening for compounds that modulate the miR content in EVs [136] may pave the way for additional, novel therapeutics for HNCs.

### 4.4. EVs and Immunotherapy

The cancer-immune cycle involves the presentation of cancer antigens by antigen-presenting cells (DCs) to induce activation of effector T cells. The activated T cells traffic to and infiltrate tumors, where cytotoxic T lymphocytes (CTLs) identify and kill cancer cells. In turn, dead cancer cells release more antigens, which repeat the above-mentioned cycle. Packaging of this content into EVs is a potentially exploitable immunotherapeutic option. There are a series of stimulatory and inhibitory factors involved in the cancer-immunity cycle. Among them, the pathway called STING, a stimulator of interferon genes, is regarded as a master regulator and mediator in each step of the cancer immunity cycle. STING is required for the presentation of cancer antigens to T-cells and is crucial for their subsequent activation [137]. In HNCs, STING activation induces type I INF in host cells and promotes a cytotoxic T cell response. In addition, STING has been shown to facilitate cetuximab mediated DC maturation and NK cell activation [138]. Since STING is ubiquitously expressed by normal cells, direct intratumoral administration of free STING agonists may result in activation of the STING pathway in all cells, resulting in the loss of viability of both immune cells and normal tissue. To overcome this issue, exoSTING were recently engineered which are EVs loaded with a STING agonist and prostaglandin F2 receptor negative regulator, a transmembrane glycoprotein that can activate antigen-presenting cells [139]. It was shown that exoSTING could be retained intratumorally (in vivo) with minimal systemic exposure and thus resulted in negligible collateral tissue damage. Compared with free STING agonist, exoSTING drove superior INFγ production and superior T-cell infiltration. Interestingly, exoSTING also caused an induction in PD-L1 expression intratumorally, which is in line with the previous observation that PD-L1 can be up-regulated by free STING [140,141]. Therefore, EV based therapy exoSTING can potentially be combined with PD-L1 immune checkpoint blockades or other targeted therapies like cetuximab and is worthy of further consideration for HNC. Additional details are now needed to enable a comprehensive evaluation of the translational potential of exoSTING.

## 5. Conclusions

We have provided evidence that HNCs use EVs in discrete ways depending on the anatomical subsite, stage, viral status, and environmental conditions. We have also discussed studies that support the notion that EV packaging is not random and is impacted by external stressors such as radiation and internal pressures such as aberrant signaling. That is, EVs can promote tumor survival, re-educate immune cells, and create pre-metastatic niches in a coordinated and deliberate fashion. Future studies are now needed to increase our understanding of the properties and mechanisms governing EV biosynthesis, trafficking, and uptake that are unique and specific for HNC to realize the full scope of their impact on disease onset and progression. There is an urgent need for future studies to address the fact that biofluid derived EVs originate from not only a complex tumor microenvironment but also non-tumor, host cells. Beyond this, an appreciation for the presence of EV subpopulations is needed to fully associate cause and function. We advocate that EVs have great potential for diagnostics and therapeutics but caution their use in HNC based on the scant literature that is currently available.

## Supplementary Materials

Supplementary materials can be found at https://www.mdpi.com/1422-0067/21/21/8260/s1; Supplementary Table S1. Summary of EV isolation and characterization.

## Abbreviations

HNC	Head and neck cancer
EVs	Extracellular Vesicles
EPs	Extracellular Particles
SCC	Squamous Cell Carcinoma
EMT	Epithelial to Mesenchymal Transition
MMP	Matrix Metalloproteinases
NPC	Nasopharyngeal Cancer
LMP1	Latent Membrane Protein 1
EBV	Epstein–Barr virus
HIF1α	Hypoxia-inducible factor 1 alpha
EGFR	Epidermal Growth Factor Receptor
HSP	Heat Shock Protein
PTEN	Phosphatase and Tensin Homolog
PD-L1	Programmed Death-1
PD-1	Programmed Death-Ligand 1
MDSC	Myeloid-Derived Stem Cells
HPV	Human Papillomavirus
DC	Dendritic Cell
EPHB2	Ephrin type B receptor 2
STAT	Signal Transducer and Activator of Transcription
VEGF	Vascular Endothelial Growth Factor
CAFs	Cancer-Associated Fibroblasts
TAMs	Tumor-Associated Macrophages
HPLFs	Human Periodontal Ligament Fibroblasts
SOCS1	Suppressor of Cytokine Signaling 1
PTEN	Phosphatase Tensin Homolog
PDCD4	Programmed Cell Death Factor 4
FOXO3a	Forkhead Box Protein O3
CSC	Cancer Stem Cell
PI3K	Phosphoinositide 3-kinase
mTOR	Mammalian Target of Rapamycin
OSCC	Oral Squamous Cell Carcinoma
CTC	Circulating Tumor Cell
cfDNA	Cell-free DNA
mtDNA	Mitochondrial DNA
dsDNA	Double-stranded DNA
ssDNA	Single-stranded DNA
miRNA	MicroRNA
lncRNA	Long Noncoding RNA
circRNA	Circular RNA
CYPA	Cyclophilin A
VCA-IgA	Viral Capsid Antigen IgA
ESCRT	Endosomal Sorting Complexes Required for Transport
CRISPR	Clustered Regularly Interspaced Short Palindromic Repeats
HER2	Human Epidermal Growth Factor Receptor 2
siRNA	Small Interfering RNA
OPMD	Oral Potentially Malignant Disorder
CTLs	Cytotoxic T Lymphocytes
